# Significance of* Gastrokine-1* Polymorphism Rs4254535 as a Prognostic Marker and its Association with Clinical Characteristics in Chinese Lung Cancer Patients

**DOI:** 10.7150/ijms.90145

**Published:** 2024-01-01

**Authors:** Zixiu Zou, Chang Xu, Zhengxing Li, Yajun Yang, Yutao Li, Zhenyu Sun, Qiang Li, Miao Li, Yuxin Chen, Gengxi Jiang, Man Xiao, Shicheng Guo, Yi Wang, Haijian Wang, Fan Xia, Yan Shang, Junjie Wu

**Affiliations:** 1 School of Life Sciences, Fudan University, Shanghai, 200438, China.; 2 Clinical College of Xiangnan University, Chenzhou, 423000, China.; 3 Department of Surgery, Navy Military Medical University Affiliated to Changhai Hospital, Shanghai, China.; 4 State Key Laboratory of Genetic Engineering, Collaborative Innovation Center for Genetics & Development, School of Life Sciences, Fudan University, Shanghai, 200438, China.; 5 School of Basic Medicine, Navy Military Medical University, Shanghai, 200433, China.; 6 Department of Respiratory and Critical Care Medicine, Shanghai East Hospital, Tongji University, Shanghai, 200120, China.; 7 Department of Pulmonary Medicine, Zhongshan Hospital, Fudan University, Shanghai, 200032, China.; 8 Nanjing Medical University, The Fourth Clinical Medical College, Nanjing, 211166, China.; 9 Department of Thoracic Surgery, Navy Military Medical University Affiliated Changhai Hospital, Shanghai, 200433, China.; 10 Department of Biochemistry and Molecular Biology, Hainan Medical University, Haikou, 571199, China.; 11 Department of Respiratory Disease, Navy 905 Hospital, Shanghai, 200235, China.; 12 Department of Respiratory and Critical Care Medicine, Shanghai Changhai Hospital, the First Afliated Hospital of Naval Medical University, Shanghai 200433, China.; 13 Department of General Medicine, the First Afliated Hospital of Naval Medical University, Shanghai 200433, China.; 14 Department of Pulmonary and Critical Care Medicine, Shanghai Geriatric Medical Center, Shanghai, 200032, China.

**Keywords:** *GKN1*, rs4254535, lung cancer, single nucleotide polymorphism, prognosis

## Abstract

**Background:** The single nucleotide polymorphism (SNP) of *Gastrokine-1* (*GKN1*) is associated with lung cancer but its association with prognosis is not clear.

**Methods:** Genomic DNA was extracted from the blood samples of 888 patients with lung cancer. The association between *GKN1* polymorphism rs4254535 and prognostic was analyzed by the Kaplan-Meier (KM) method, Log-rank test, and Cox proportional hazards model.

**Results:** In females and patients diagnosed with late-stage lung cancer, the CC genotype (CC vs TT, adjusted odds ratio [HR] = 0.57, 95% Confidence Interval [CI]: 0.33-0.99, P = 0.045; HR = 0.66, 95% CI: 0.48-0.92, P = 0.014) and recessive CC genotype (CC vs TT + TC, HR = 0.55, 95% CI: 0.32-0.94, P = 0.028; HR = 0.64, 95% CI: 0.47-0.89, P = 0.006) of rs4254535 conferred a better prognosis, compared with the TT and TT + TC genotype. Rs4254535 dominate TC + CC genotype, recessive CC genotype, and C allele who were adenocarcinoma patients had a significantly better prognosis. The recessive CC genotype of non-smoking patients has a better prognosis, compared to the TT + TC genotype. Additionally, in the dominant TT + TC genotype and C allele, no family history patients had a significantly better prognosis, compared to the TT genotype.

**Conclusion:** For lung cancer patients, *GKN1* polymorphism rs4254535 may be a protective genetic marker and predicts the prognosis of lung cancer patients.

## Introduction

Lung cancer is the second most commonly diagnosed cancer worldwide (11.4%), and remains the leading cause of cancer death (18%), which poses a serious threat to the life span and living quality of people 1, 2. Lung cancer is classified into Small Cell Lung Cancer (SCLC) and Non-Small Cell Lung Cancer (NSCLC) according to histopathology 3. SCLC is the most invasive lung cancer histopathological subtype, accounts for 13-15% of new lung cancer, and has less than 7% 5-year survival rate 4, 5. NSCLC accounts for about 85% of new lung cancer cases. Although there has been a tremendous advance in the treatment of NSCLC, the overall survival rate remains low 6. The complication of metastatic tumors is the critical factor in most deaths associated with lung cancer 7, 8. In addition, genetic polymorphisms and clinical characteristics significantly affect the prognosis of lung cancer patients.

*GKN1* is also called Antrum Mucosal Protein (AMP)-18, which is secreted by mucus cells on the surface of the gastric antrum and base and has growth factor or cytokine-like activity on gastric epithelial cells 9. Studies have shown that expression of* GKN1* can regulate cell proliferation and differentiation 10-12, up-regulation of GKN1 can inhibit gastric epithelial cells developing into cancer cells by down-regulating NF-κB expression 13-20 and overexpression of GKN1 promotes lung cancer cell metastasis *in vitro* 21. A high level of GKN1 protein, with which Keratin 14 (K14) - high cancer cells could resist anoikic, plays a vital role in promoting metastasis of lung cancer cells with low K14 expression. Meanwhile,* GKN1* is a specific up-regulated gene in adenocarcinoma 22. As a common genetic variation, Single nucleotide polymorphism (SNP) proves to be associated with the prognosis of lung cancer 23-30. The rs4254535 is about 3 Kbp upstream of *GKN1* and 20 Kbp upstream of *GKN2*, which are located on a pair of complementary chains. Previous studies have found that the *GKN1* polymorphism rs4254535 is related to the chemotherapy response of lung cancer patients. A study on the Chinese Han population find that compared with "TT" genotype carriers, "CT" carriers have worse responses to cisplatin-based chemotherapy 31.

To further study whether *GKN1* polymorphism rs4254535 is associated with the prognosis of lung cancer, we collected 888 blood samples from patients diagnosed with lung cancer. The blood samples were taken before treatment to detect* GKN1* polymorphism rs4254535 and a follow-up investigation was conducted to explore the association between *GKN1* polymorphism rs4254535 and disease progression and prognosis of patients with various types of lung cancer. The results are based on accurate data and rigorous statistical analysis.

## Patients and methods

### Study Group

From January to November 2009, a total of 888 patients with primary lung cancer were included, and 839 cases were retained after excluding 49 cases of incomplete data. The follow-up period ended in 2019. The patients in this cohort were from Changhai Hospital Affiliated to the Naval Military Medical university (Second Military Medical University) (n = 536) and Taizhou Institute of Health Sciences of Fudan University (n = 352). Eligibility criteria: Primary lung cancer diagnosed by histopathological diagnosis, no history of other malignancy, no age and sex restrictions. Patient clinical data were obtained based on patient history and telephone follow-up. The study was approved by the Ethics Committee of the School of Life Sciences of Changhai University, and all the subjects received informed consent.

### SNP Genotyping

All selected patients collected 5 ml blood samples before treatment, genomic DNA was extracted using the QIAamp DNA Blood Mini Kit (Qiagen, 51106) and genotyping was performed using the 2×48-plex SNPscan TM Kit (Genesky Biotechnologies, G0104) 32, 33. We used a detailed procedure to determine genotyping quality, guaranteed a call rate of more than 99%, reproduced genotype detection, internal positive control samples, and performed Hardy-Weinberg equilibrium (HWE) testing. Genotype analysts were blinded to the patients' clinical information.

### Statistical analysis

Pearson's chi-square test was used to check for Hardy-Weinberg equilibrium (HWE). Overall survival (OS) was calculated from the date of sample collection until the date the patient died from any cause or the last time of follow-up visit. In this study, the median survival time (MST) was estimated using the Kaplan-Meier (KM) method, the Log-rank test compared the differences between groups, and the multivariate Cox proportional hazards model estimated the hazard ratio (HR) and 95% confidence interval (CI) of age and sex adjustment. Four SNP genetic models (allele, genotype, dominant, and recessive) were analyzed. The data were also analyzed by age, sex, smoking status, family history of lung cancer, histological type of lung cancer, and TNM stage. The statistical significance was considered at P < 0.05. All respective tests were bilateral and performed using R version 3.6.2 (Vienna, Austria).

## Results

### Demographic and general clinical Characteristics

The follow-up period was from the enrollment until November 15, 2019. Due to incomplete data, 49 patients were excluded, and the remaining 839 patient data were analyzed. The study sample was an ethnically homogeneous group of Han Chinese individuals. A total of 668 deaths (79.6%), 103 (12.3%) survived for more than 5 years, 68 cases (8.1%) were lost follow-up, 610 males (72.7%), 524 (62.5%) were aged ≥ 60 years, 582 (69.4%) had smoking history, 537 (64%) had no family history of malignant tumors, 154 (18.4%) and 625 (74.5%) were staged I-II and III-IV respectively. As for the cancer subtypes, 367 (43.7%) patients were diagnosed with adenocarcinoma, 282 (33.6%) with squamous cell carcinoma (SCC), 72 (8.6%) with small cell lung cancer (SCLC), and 118 (14.1%) with other cancer types, including adenosquamous carcinoma (ASC), large cell carcinoma (LCC), carcinosarcoma (CS) and mucoepidermoid carcinoma (MEC) (**Table 1**).

### Association between patients' Characteristics and lung cancer outcomes

MST varied from a few months (M) to tens of months across different stratifications. As shown in **Table 1**, the MST was much lower for men than for women (34.27 vs. 40.17 M; P = 0.01), The MST of patients aged 60 and over is much lower than that of patients under 60. (33.2 vs. 40.87 M; P = 0.003), smokers were much lower than non-smokers (33.9 vs. 41.03 M; P < 0.001), patients with advanced tumor stage were much lower than those with early-stage tumors (29.4 vs. 113.93 M; P < 0.001).

### Association between *GKN1* polymorphism rs4254535 and lung cancer prognosis

A total of 839 genotypes were detected, with a call rate of 99.64%, of which 439 were TT, 329 TC, and 68 CC genotypes. Among all the patients who died, the proportion of genotype TT and dominant TT + TC were 52.03% (346/665) and 92.48% (615/665), respectively, which were significantly different from CC genotype (P = 0.02), suggesting that the latter had a lower prognosis. The MST of the CC genotype was 47.43 M, which was much higher than that of the former two at 33.63 M and 34.73 M (**Table 2**).

### Association of *GKN1* polymorphism with lung cancer prognosis

**Figure 1:**
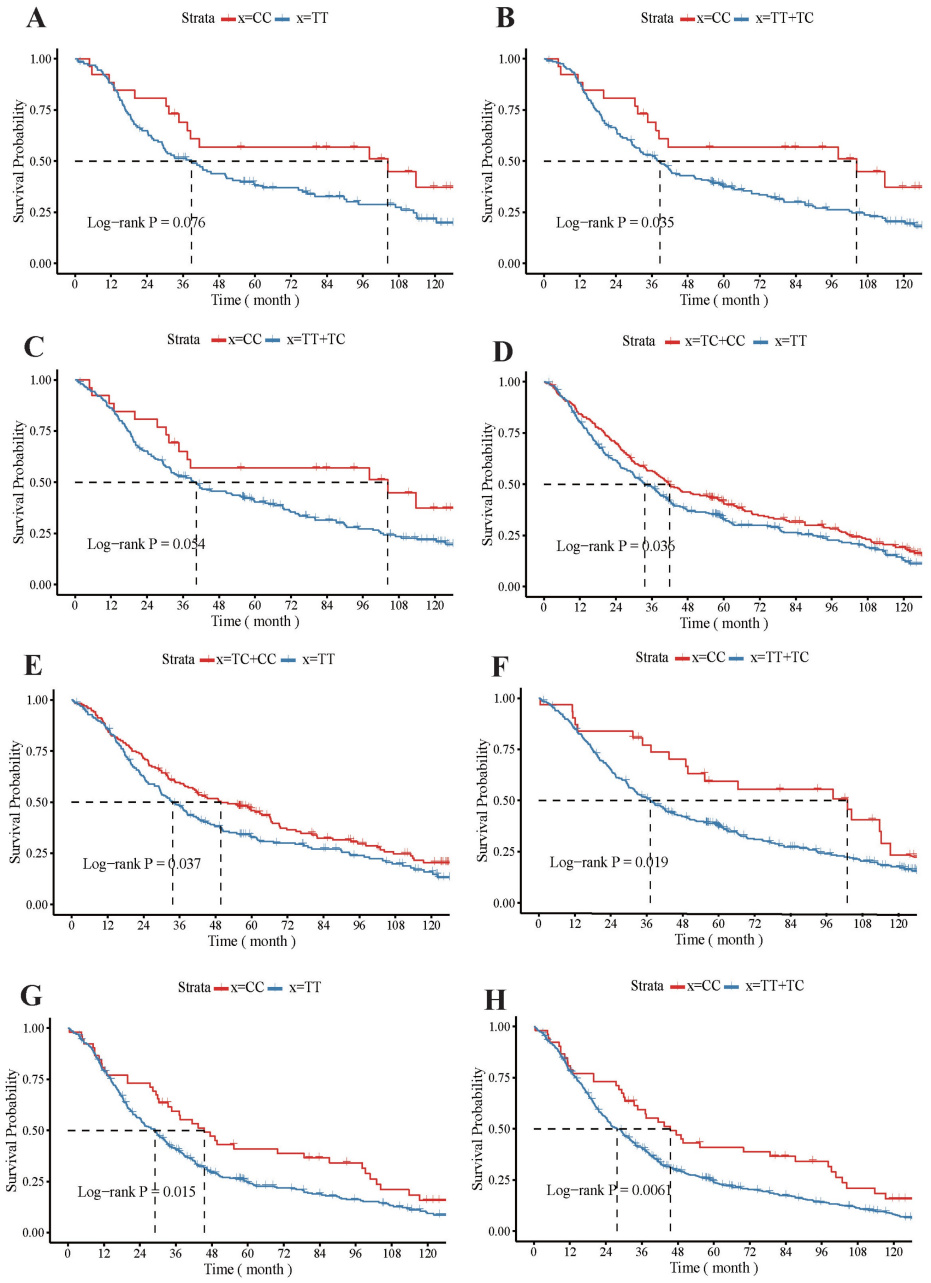
Impacts of *GKN1* polymorphism rs4254535 on the prognosis of patients with lung cancer patients. The Kaplan-Meier survival curve analysis of the *GKN1* polymorphism rs4254535 and TT genotype (**A**) and the recessive TT + TC genotype in female (**B**), the CC genotype non-smoker patients (**C**), and no family history patients with dominant TC + CC genotype (**D**) survival probability. Impacts of *GKN1* polymorphism rs4254535 on the prognosis of patients with dominate TT + TC genotype (**E**) and CC genotype (**F**) who adenocarcinoma patients, the CC genotype (**G**) and recessive TT+TC genotype (**H**) patients with late-stage (stage III + IV) lung cancer.

The KM survival curve showed that the rs4254535 polymorphism T > C could significantly increase the MST of several kinds of patients. For example, the Kaplan-Meier curves demonstrated that MST was longer in female patients with the CC (MST: 104.3 M) genotype compared to those with the TT (MST: 38.77 M) genotype (Log-rank P = 0.076;** Figure 1 A**), and MST was longer in the CC genotype (MST: 104.3 M) who female patients, compared to the recessive TT + TC (MST: 38.77 M) genotype (Log-rank P = 0.035; **Figure 1 B**). For non-smoker patients with CC genotype (MST: 104.3 M; Log-rank P = 0.054; **Figure 1 C**), their median survival time was higher than that with recessive TT + TC genotype (MST: 40.4 M). In no family history patients, the dominant TC + CC genotype (MST: 41.9 M) could also highly improve survival rate compared with the TT genotype (MST: 33.63 M; Log-rank P = 0.036146; **Figure 1 D**). Additionally, in the dominant TT + TC genotype (MST: 49.77 M) adenocarcinoma patients had a significantly better MST, compared to the TT genotype (MST: 33.63M; Log-rank P = 0.037; **Figure 1 E**), and MST was longer in the CC genotype (MST: 102.9 M) who adenocarcinoma, compared to the recessive TT + TC genotype (MST: 37.17 M; Log-rank P = 0.019; **Figure 1 F**). In patients with late-stage (stage III + IV) lung cancer, the MST was significantly longer in patients with the CC genotype (MST: 45.5 M) than TT genotype (MST: 37.17 M; Log-rank P = 0.015; **Figure 1 G**), and CC genotype (MST: 45.5 M) of MST compared to the recessive TT+TC genotype longer (MST: 26.3 M; Log-rank P = 0.006; **Figure 1 H**).

### Association between *GKN1* polymorphism rs4254535 and lung cancer prognosis stratified by patients' characteristics

The *GKN1* rs4254535 allele C significantly increased the prognosis of no family history patients of lung cancer (MST: 42.3 M; P = 0.0281), and adenocarcinoma (MST: 55.37 M; P = 0.008) patients, and effectively prolonged MST (**Table 3**). Compared with the TT genotype, the CC genotype had a significantly better prognosis of females (P = 0.045), and patients diagnosed with late-stage lung cancer (P = 0.014) (**Table 4**). Compared with the TT genotype, the dominant TC + CC genotype had a better effect on the prognosis of patients without a family history (P = 0.046) and significantly increased the median survival time of patients with adenocarcinoma (P = 0.0396; MST: TT, 33.63 M; TC + CC, 49.77 M; **Table 5**). Compared with the recessive TT + TC genotype, the CC genotype in non-smoke (MST: 104.3 M; P = 0.050) and late-stage patients (MST: 45.5 M; P = 0.0069) improved prognosis and significantly prolonged MST, particularly in patients with adenocarcinoma (MST: 102.9 M; P = 0.0255; **Table 6**).

## Discussion

The research investigated the association between the prognosis and the *GKN1* polymorphism rs4254535 by using blood samples from 839 Chinese patients with lung cancer. The authors observed that rs4254535 polymorphism T > C, an SNP in *GKN1*, was significantly associated with better prognosis in female, non-smoking, No Family History, and adenocarcinoma patients diagnosed with late-stage (stage III + IV) lung cancer.

Expression of GKN can be used as a good prognostic marker and therapeutic target for lung and stomach cancer, and the OS rate of patients with low GKN2 mRNA expression is poor 34, 35. The ectopic expression of *GKN1* can inactivate the NF-κB signaling pathway and activate lκB to increase GKN2 expression by time-dependent means 36. *GKN1* also has a clear anti-proliferative effect and plays the role of antitumor 37. In addition, in the development of gastric cancer in humans and mice, the highly coordinated deletion of *GKN1/GKN2* genes and the abnormal expression (deletion) of GKN are closely related to the occurrence of precancerous inflammation and gastric cancer 38. In a study on the effect of *GKN1* polymorphism on the efficacy of cisplatin chemotherapy in lung cancer, it has been found that* GKN1* polymorphism significantly reduce the chemotherapy response of lung cancer patients 39. These studies have shown that *GKN* polymorphism or loss of expression is closely related to the occurrence and development of cancer. In our study, *GKN1* polymorphism rs4254535 was found to be significantly associated with lung cancer prognosis, especially in adenocarcinoma and non-smoking patients.

Sex and cancer clinical stage are important clinical characteristics that affect the prognosis of lung cancer patients, and the difference in prognosis due to gender are mainly closely related to estrogen, the effect of estrogen is more obvious in women and is an important biological factor leading to lung cancer 40, 41. In addition, female cancer patients generally have lower morbidity and mortality compared with males, mainly due to higher expression of tumor suppressor genes encoded by the X chromosome 42. At the same time, gender synergistic genetic polymorphisms significantly affect the occurrence and development of lung cancer patients 43. However, another study has found women have a higher prevalence of lung cancer than men, because of the high expression of *Cytochrome P-450 1A1(CYPlA1)* and *P53* genes, which are genetically susceptible to tobacco and airborne pollutants 44. A study of 36,658 patients with primary lung cancer in West China Hospital from 1995 to 2015 also finds that the incidence and number of lung cancer in women increased year by year, especially in adenocarcinoma 45. The clinical stage is also an important clinical feature that differentiates cancer prognosis. In a study on the association between TNFRSF19 expression and lung cancer risk, it has been found that the differentiated expression of TNFRSF19 is statistically significantly associated with tumor TNM stage and patient survival 46. In our study, *GKN1* polymorphism rs4254535 was significantly associated with female and better prognosis in patients with advanced lung cancer.

In stratified analysis, we observed that *GKN1* polymorphism rs4254535 was significantly associated with better prognosis in lung cancer patients with no smoking history and those diagnosed with lung adenocarcinoma cancer. Many compounds in tobacco smoke, such as nicotine, are classified as lung carcinogens and have proven to be closely related to the development and prognosis of lung cancer 47, 48. Through the collation and analysis of 383 research literature on the association between smoking and lung cancer in the past 20 years, the study has found that smoking can affect the proliferation and differentiation of lung cancer cells by affecting the acetylcholine system and thus affect the progression of lung cancer 49. Smoking can also affect gene expression through epigenomic modification and thus affect lung cancer prognosis 50. Genetic polymorphisms cooperate with smoking to significantly affect the prognosis of lung cancer patients. In a study on the impact of genetic polymorphisms and smoking on the prognosis of lung cancer patients, it is found that genetic polymorphisms synergize with smoking to significantly reduce patient survival prognosis 51. In addition, another important factor affecting prognosis is cancer tissue type. Multiple studies have found that patients diagnosed with adenocarcinoma significantly change their prognosis under the influence of genetic polymorphisms 52, 53. Our study also found that the rs4254535 genetic polymorphism significantly increases the prognosis of patients with lung adenocarcinoma.

The association of genetic tools and clinical characteristics of specific cancers is giving medicine a new focus by means of “personalized cancer drugs” 54, 55. A new and effective biomarker gives a new target for personalized cancer drug treatment, helps to select appropriate individualized patients to evaluate target regulation, and guides evidence-based treatment of early disease, and it is valuable in improving the prognosis of cancer patients 56. Moreover, strengths of this study, we tested all lung cancer patients for polymorphisms in known genetic loci, reduced the possibility of missing important genomic possibilities as well as avoided data complexity. In addition, there were certain limitations in our study: only the association analysis between the rs4254535 single locus and lung cancer prognosis on the *GKN1* gene, which lacked the exploration of genome-wide interactions 57. Cancer prognosis is related to several factors 58, but we did not involve the influence of psychological and social factors on the progression and prognosis of patients' diseases in our study.

## Conclusions

The study showed that *GKN1* polymorphism rs4254535 T > C significantly reduced the prognosis of lung cancer patients and was relatively strongly associated with non-smoking, no family history, female, and adenocarcinoma patients. Although there were still some deficiencies in this study, it still might have potential clinical significance for the prognosis and treatment strategy of lung cancer patients. Patients with poor prognosis, such as non-smokers, females, and no family history of cancer, should undergo lung cancer testing for clinical characteristics identification and genetic polymorphisms. Based on different clinical characteristics and specific nucleotide site polymorphisms, they should receive personalized high-quality treatment as early as possible, and improve prognosis. In addition, our research model provides a new target for the treatment of lung cancer at the rs4254535 nucleotide site.

## Figures and Tables

**Table 1:** Characteristic distribution in Chinese patients with lung cancer and prognosis analysis.

Variables	N (%)	MST	Log-rank *P*
All	839	36.73	
Sex			0.01
Female	229 (27.3 %)	40.17	
Male	610 (72.7 %)	34.27	
Age			0.003
Age < 60	315 (37.5 %)	40.87	
Age >= 60	524 (62.5 %)	33.2	
Smoking status			< 0.001
Nonsmoker	237 (28.2 %)	41.03	
Smoker	582 (69.4 %)	33.9	
unknown	20 (2.4 %)	67	
Family History			0.462
Yes	302 (36 %)	33.63	
No	537 (64 %)	38.03	
Stage			< 0.001
Early stage	154 (18.4 %)	113.93	
Late stage	625 (74.5 %)	29.4	
Unknown	60 (7.2 %)	66.43	
Subtype			0.211
ADC	367 (43.7 %)	38.8	
SCC	282 (33.6 %)	33.63	
SCLC	72 (8.6 %)	33.9	
Other*	118 (14.1 %)	36.2	

^*^ Other carcinomas include ASC, LCC, CS and MEC.

**Table 2:** Association analysis between *GKN1* polymorphism rs4254535 and prognosis of lung cancer patients.

SNP	Model	Death/survival	MST	HR (Cl)	*P*	HR^*^ (95% CI)	*P* ^*^
rs4254535	Allele						
	T (ref)	961/246	34.27	1		1	
	C	369/96	39.50	0.91 (0.80-1.02)	0.105	0.90 (0.80-1.02)	0.100
	Genotype						
	T/T (ref)	346/93	33.63	1		1	
	T/C	269/60	37.10	0.97 (0.83-1.14)	0.754	0.96 (0.82-1.12)	0.593
	C/C	50/18	47.43	0.74 (0.55-1.00)	0.049	0.76 (0.56-1.02)	0.065
	Dominate						
	T/T (ref)	346/93	33.63	1		1	
	T/C+C/C	319/78	38.5	0.93 (0.80-1.08)	0.345	0.92 (0.79-1.07)	0.272
	Recessive						
	T/T+T/C (ref)	615/153	34.73	1		1	
	C/C	50/18	47.43	0.75 (0.56-1.00)	0.052	0.77 (0.58-1.03)	0.077

^*^ CI, confidence interval; HR, hazard ratio; ref, reference. ^a^ Adjusted by age, sex.

**Table 3:** Association between *GKN1* polymorphism rs4254535 in allele model and prognosis in Chinese patients with lung cancer.

Variables	Death/survive		HR (95% CI)	*P*	HR^*^ (95% CI)	P^*^
	T (ref)	C				
Sex						
Female	243/89	89/37	0.84 (0.66-1.07)	0.159	0.82 (0.65-1.05)	0.118
Male	718/157	280/59	0.93 (0.81-1.07)	0.306	0.93 (0.81-1.07)	0.322
Age						
Age < 60	339/122	121/46	0.91 (0.74-1.12)	0.358	0.86 (0.69-1.06)	0.146
Age >= 60	622/124	248/50	0.91 (0.78-1.05)	0.195	0.91 (0.78-1.06)	0.214
Smoking status						
Nonsmoker	247/96	91/40	0.82 (0.64-1.04)	0.097	0.81 (0.64-1.04)	0.096
Smoker	694/139	274/51	0.94 (0.82-1.08)	0.408	0.95 (0.82-1.09)	0.372
Family History						
Yes	336/80	152/30	1.04 (0.86-1.26)	0.690	1.03 (0.85-1.25)	0.775
No	625/166	217/66	0.82 (0.71-0.96)	0.014	0.84 (0.72-0.98)	0.028
Stage						
Early stage	117/107	35/49	0.79 (0.54-1.15)	0.216	0.79 (0.54-1.16)	0.234
Late stage	771/131	303/39	0.91 (0.80-1.04)	0.160	0.90 (0.79-1.03)	0.135
Subtype						
ADC	407/123	143/59	0.77 (0.63-0.93)	0.006	0.77 (0.64-0.94)	0.008
SCC	331/65	145/21	1.06 (0.87-1.29)	0.545	1.06 (0.87-1.28)	0.587
SCLC	86/20	30/6	0.75 (0.49-1.15)	0.188	0.73 (0.47-1.13)	0.156
NSCLC	875/226	339/90	0.92 (0.81-1.04)	0.190	0.92 (0.81-1.04)	0.198

^*^ Adjusted by age, gender. CI, confidence interval; HR, hazard ratio; ref, reference.

**Table 4:** Association between *GKN1* polymorphism rs4254535 in genotype models and prognosis of Chinese patients with lung cancer.

Variables	Death/Survive			T/C vs T/T		C/C vs T/T	
	T/T (ref)	T/C	C/C	HR^*^ (95% Cl) P^*^		HR^*^ (95% Cl) P^*^	
Sex							
Male	254/56	210/45	35/7	0.92 (0.77-1.10)	0.370	0.88 (0.62-1.25)	0.477
Female	92/37	59/15	15/11	1.11 (0.80-1.54)	0.547	0.57(0.33-0.99)	0.045
Age(year)							
Age < 60	126/47	87/28	17/9	0.96(0.73-1.26)	0.778	0.76 (0.46-1.27)	0.301
Age >= 60	220/46	182/32	33/9	0.98 (0.80-1.19)	0.806	0.77 (0.53-1.11)	0.160
Smoking status							
Yes	245/51	204/37	35/7	0.97(0.80-1.16)	0.711	0.87 (0.61-1.24)	0.427
No	93/39	61/18	15/11	-	-	0.58 (0.33-1.01)	0.056
Family cancer history							
Yes	116/30	104/20	24/5	1.22(0.93-1.61)	0.144	0.88 (0.57-1.38)	0.588
No	230/63	165/40	26/13	0.85(0.69-1.04)	0.134	0.68(0.45-1.03)	0.065
Histological type							
ADC	151/44	105/35	19/12	0.84 (0.66-1.09)	0.189	0.55 (0.34-0.88)	0.013
SCC	110/25	111/15	17/3	1.03 (0.79-1.35)	0.810	1.20 (0.72-1.99)	0.494
SCLC	32/9	22/2	4/2	1.06 (0.61-1.83)	0.841	0.33(0.10-1.12)	0.076
NSCLC	314/84	247/58	46/16	0.95 (0.80-1.13)	0.561	0.81 (0.59-1.10)	0.179
staging							
Early tage	45/34	27/39	4/5	0.65 (0.40-1.05)	0.078	0.98 (0.35-2.73)	0.963
Late stage	275/57	221/17	41/11	1.08 (0.90-1.29)	0.426	0.66 (0.48-0.92)	0.014

^*^ Adjusted by age, gender. CI, confidence interval; HR, hazard ratio; ref, reference.

**Table 5:** Association between *GKN1* polymorphism rs4254535 in dominant genotype and prognosis of Chinese patients with lung cancer.

Variables	Death/survive		HR (95% CI)	*P*	HR^*^ (95% CI)	P^*^
	T/T (ref)	T/C + C/C				
Sex						
Female	92/37	74/26	0.95 (0.70-1.29)	0.731	0.93 (0.68-1.27)	0.647
Male	254/56	245/52	0.91 (0.76-1.09)	0.297	0.91 (0.77-1.09)	0.314
Age						
Age < 60	126/47	104/37	0.93 (0.72-1.21)	0.587	0.92 (0.71-1.20)	0.545
Age >= 60	220/46	215/41	0.93 (0.77-1.12)	0.450	0.94 (0.77-1.13)	0.487
Smoking stage						
Nonsmoker	93/39	76/29	-	-	-	-
Smoker	245/51	239/44	0.95 (0.79-1.13)	0.536	0.95 (0.79-1.14)	0.569
Family History						
Yes	116/30	128/25	1.14 (0.89-1.47)	0.295	1.14 (0.88-1.47)	0.325
No	230/63	191/53	0.81 (0.67-0.99)	0.036	0.82 (0.68-1.00)	0.046
Stage						
Early stage	45/34	31/44	0.68 (0.43-1.08)	0.102	0.68 (0.43-1.08)	0.100
Late stage	275/57	262/28	0.99 (0.84-1.18)	0.931	0.98 (0.83-1.16)	0.817
Subtype						
ADC	151/44	124/47	0.78 (0.61-0.98)	0.037	0.78 (0.61-0.99)	0.039
SCC	110/25	128/18	1.07 (0.83-1.38)	0.598	1.05 (0.81-1.36)	0.694
SCLC	32/9	26/4	0.89 (0.53-1.51)	0.671	0.86 (0.50-1.46)	0.574
NSCLC	314/84	293/74	0.93 (0.80-1.09)	0.393	0.93 (0.79-1.09)	0.339

^*^ Adjusted by age, gender. CI, confidence interval; HR, hazard ratio; ref, reference.

**Table 6:** Association between *GKN1* polymorphism rs4254535 in recessive genotype and prognosis of Chinese patients with lung cancer.

Variables	Death/survive		HR (95% CI)	*P*	HR^*^ (95% CI)	P^*^
	T/T + T/C (ref)	C/C				
Sex						
Female	151/52	15/11	0.57 (0.33-0.97)	0.037	0.55 (0.32-0.94)	0.028
Male	464/101	35/7	0.91 (0.65-1.29)	0.597	0.91 (0.65-1.29)	0.610
Age						
Age < 60	213/75	17/9	0.76 (0.46-1.25)	0.283	0.78 (0.47-1.28)	0.320
Age >= 60	402/78	33/9	0.75 (0.53-1.08)	0.118	0.78 (0.54-1.11)	0.166
Smoking stage						
Nonsmoker	154/57	15/11	0.60 (0.35-1.01)	0.056	0.58 (0.34-1.00)	0.050
Smoker	449/88	35/7	0.87 (0.61-1.22)	0.414	0.88 (0.62-1.24)	0.468
Family History						
Yes	220/50	24/5	0.84 (0.55-1.28)	0.409	0.81 (0.53-1.25)	0.345
No	395/103	26/13	0.68 (0.46-1.02)	0.059	0.73 (0.49-1.09)	0.124
Stage						
Early stage	72/73	4/5	1.09 (0.40-3.00)	0.860	1.18 (0.43-3.23)	0.751
Late stage	496/74	41/11	0.64 (0.47-0.88)	0.006	0.64 (0.47-0.89)	0.006
Subtype						
ADC	256/79	19/12	0.58 (0.36-0.92)	0.020	0.59 (0.37-0.94)	0.025
SCC	221/40	17/3	1.14 (0.70-1.87)	0.602	1.18 (0.72-1.93)	0.519
SCLC	54/11	4/2	0.34 (0.11-1.10)	0.072	0.32 (0.10-1.07)	0.064
NSCLC	561/142	46/16	0.80 (0.59-1.08)	0.149	0.83 (0.61-1.12)	0.214

^*^ Adjusted by age, gender. CI, confidence interval; HR, hazard ratio; ref, reference.

## Abbreviations

SNP: single nucleotide polymorphism
*GKN1*: *Gastrokine-1*
KM: Kaplan-Meier
CI: Confidence Interval
ADC: adenocarcinoma
SCLC: Small Cell Lung Cancer
NSCLC: Non-Small Cell Lung Cancer
AMP: Antrum Mucosal Protein
K14: Keratin 14
HWE: Hardy-Weinberg equilibrium
OS: Overall survival
MST: Median Survival Time
HR: hazard ratio
TNM: Tumor Node Metastasis
SCC: squamous cell carcinoma
ASC: adenosquamous carcinoma
LCC: large cell carcinoma
CS: Carcinosarcoma
MEC: mucoepidermoid carcinoma
M: months
*CYPlA1*: *cytochrome P-450 1A1*
